# Stable clinical course in three siblings with late-onset isolated sulfite oxidase deficiency: a case series and literature review

**DOI:** 10.1186/s12887-019-1889-5

**Published:** 2019-12-23

**Authors:** Maoqiang Tian, Yi Qu, Lingyi Huang, Xiaojuan Su, Shiping Li, Junjie Ying, Fengyan Zhao, Dezhi Mu

**Affiliations:** 1 Department of Pediatrics, West China Second University Hospital, Sichuan University, Chengdu, 610041 China; 2 Key Laboratory of Birth Defects and Related Diseases of Women and Children, (Sichuan University), Ministry of Education, Chengdu, 610041 China; 3 Department of Pediatrics, Affiliated Hospital of Zunyi Medical University, Zunyi, 563003 China; 4 West China College of Stomatology, Sichuan University, Chengdu, 610041 China

## Abstract

**Background:**

Isolated sulfite oxidase deficiency (ISOD) is an autosomal recessive disorder caused by a deficiency of sulfite oxidase, which is encoded by the sulfite oxidase gene (*SUOX*). Clinically, the disorder is classified as one of two forms: the late-onset mild form or the classic early-onset form. The latter is life-threatening and always leads to death during early childhood. Mild ISOD cases are rare and may benefit from dietary therapy. To date, no cases of ISOD have been reported to recover spontaneously. Here, we present three mild ISOD cases in one family, each with a stable clinical course and spontaneous recovery.

**Case presentation:**

All three siblings had two novel compound heterozygous mutations in the *SUOX* gene (NM_000456; c.1096C > T [p.R366C] and c.1376G > A [p.R459Q]). The siblings included two males and one female with late ages of onset (12–16 months) and presented with specific neuroimaging abnormalities limited to the bilateral globus pallidus and substantia nigra. The three patients had decreased plasma homocysteine levels. They exhibited a monophasic clinical course continuing up to 8.5 years even without dietary therapy.

**Conclusion:**

This is the first report of mild ISOD cases with a stable clinical course and spontaneous recovery without dietary therapy. Our study provides an expansion for the clinical spectrum of ISOD. Furthermore, we highlight the importance of including ISOD in the differential diagnosis for patients presenting with late-onset symptoms, bilaterally symmetric regions of abnormal intensities in the basal ganglia, and decreased plasma homocysteine levels.

## Background

Isolated sulfite oxidase deficiency (ISOD, OMIM 272300) is an inborn error of metabolism characterized by severe neurological impairment including dystonia severe psychomotor delay, refractory seizures, and lens dislocation. Clinically, the disorder is classified as one of two forms: mild or severe. Severe ISOD often presents within the first 72 h of life and leads to death during early childhood in most cases [1, 2]. ISOD is caused by a mutation in the sulfite oxidase gene (*SUOX*) [2]. Sulfite oxidase (SUOX) is located in the intermembrane space of the mitochondria and catalyzes the degradation of sulfur-containing amino acids [3–5]. Mutations in the *SUOX* gene result in a decrease in SUOX activity, leading to an accumulation of sulfur-containing amino acids, inducing mitochondrial impairment and energy failure, and resulting in severe neurological damage [2]. Effective treatment is not available for most patients with ISOD [6], but clinical and biochemical improvements were observed in six mild ISOD cases treated with dietary therapy involving restricting the intake of methionine, cysteine, and taurine [7–11]. Therefore, early identification of ISOD, especially the mild type, may allow patients to benefit from dietary therapy. To date, no cases of ISOD have been reported to recover spontaneously. We report three cases of mild ISOD which experienced spontaneous recovery and maintained a stable clinical course even 8.5 years into follow-up.

## Case presentation

**Fig. 1:**
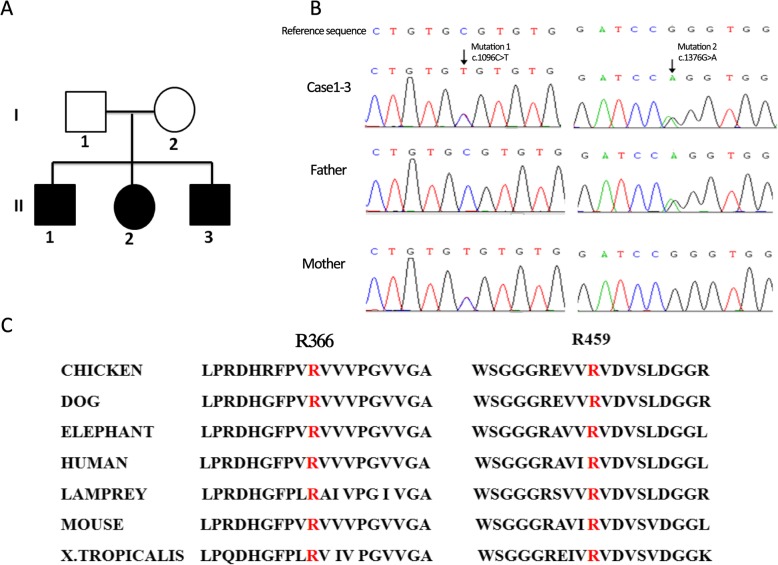
Genetic analysis of *SUOX* in our family. **a**, Family pedigree with mutations in *SUOX* gene. Filled: affected with ISOD (compound heterozygous mutation); not filled, mutation carrier (heterozygous mutation). **b**, Sequencing electropherograms of all three affected siblings showing c.1096C > T (mutation 1, maternal) and c.1376G > A (mutation 2, paternal) transition. **c**, Phylogenetic conservation of the R366 and R459 (highlighted in red). These residues were conserved between species during evolution

The three patients in this report were children of a non-consanguineous Chinese couple (Fig. 1a) who were born at term and followed a normal delivery. Their psychomotor developmental milestones were normal prior to disease onset. The main biochemical findings of each patient are summarized in Table 1**.** This study was approved by the Ethics committee of the Affiliated Hospital of Zunyi Medical University, China.

**Table 1:** Main biochemical findings of the three patients in this study with late-onset mild ISOD

Parameter	Case 1	Case 2	Case 3	Normal value
Urine sulfite reaction (age)	Positive (9 years)	Positive (5 years)	Positive(4 years)	Negative
Urine sulfite level (age)	45 mg/L (9 years) 50 mg/L (9 years and 3 months)	40 mg/L (5 years) 30 mg/L (5 years and 3 months)	100 mg/L (4 years) 25 mg/L(4 years and 3 months)	< 15 mg/L
Homocysteine (age)	3.74 μmol/L (9 years)	3.17 μmol/L (5 years)	2.48 μmol/L (16 months) 2.66 μmol/L (4 years)	5-15 μmol/L
Uric acid (age)	255 μmol/L (1 year)	269 μmol/L (14 months)	400 μmol/L (16 months)	208–428 μmol/L
Blood lactate (age)	2.4 mmol/L (1 year)	5 mmol/L (14 months)	2.48 mmol/L (16 months)	< 2 mmol/L
Cerebrospinal fluid results				
Cell count (age)	10 cells/μl (1 year)	5 cells/μl (14 months)	8 cells/μl(16 months)	< 10cells/μl
Protein (age)	342 mg/L (1 year)	159 mg/L (14 months)	269 mg/L(16 months)	< 450 mg/L
Glucose (age)	3.2 mmol/L (1 year)	3.44 mmol/L (14 months)	2.95 mmol/L(16 months)	> 2.8 mmol/L

### Case 1

**Fig. 2:**
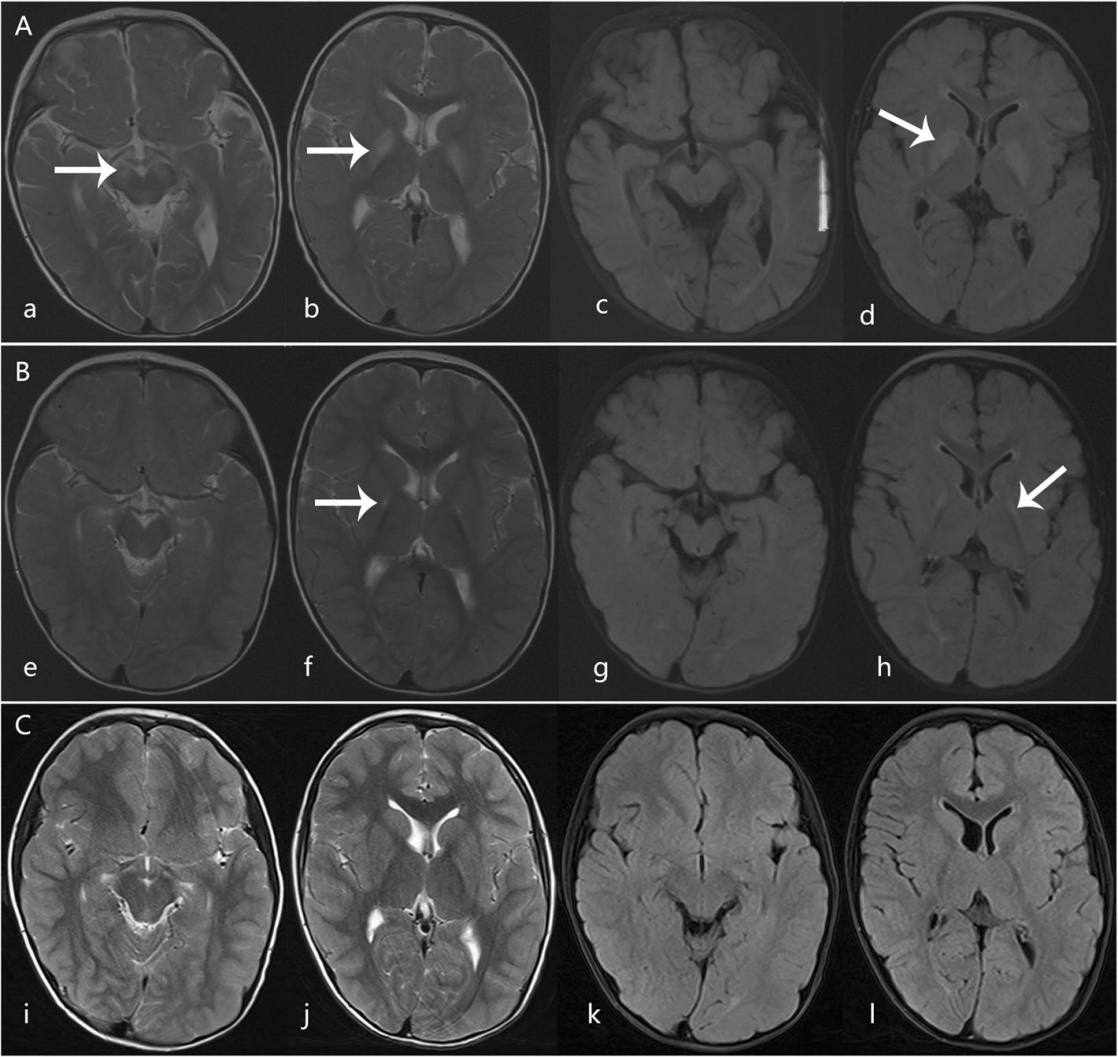
Axial T2-weighted imaging (T2WI) and fluid-attenuated inversion recovery (FLAIR) magnetic resonance imaging (MRI) scans from Case 1. **a**, **b**, T2 W1 and **c**, **d**, FLAIR performed 3 days after disease onset at age 1, showing high signal in the bilateral globus pallidus and substantia nigra (arrows). **e-h**, Follow-up images 1 year after onset revealed that hyper-signal lesions significantly shrank (arrows). **i-l**, Lesions on the basal ganglia had almost disappeared and the lesion on the substantia nigra disappeared 5.5 years after the onset

Before age 1, psychomotor development in the firstborn male patient was normal, and he could walk independently. This patient exhibited rapid regression of acquired motor skills and cognition after a 2-day episode of mild diarrhea at age 1. Seizures were not observed. On examination, his growth was found to be in the 90th percentile, and his alertness and eye movement were both normal. He was not able to sit. No dysmorphic features, tremor, ataxia, or involuntary movements were observed. Cranial nerve examination findings were normal. He had generalized hypotonia with normal deep tendon reflexes. His blood lactate level was slightly increased, but uric acid, plasma glucose, and cerebrospinal fluid (CSF) test results were unremarkable. Brain magnetic resonance imaging (MRI) using T2-weighted (T2WI) and fluid-attenuated inversion recovery (FLAIR) sequencing demonstrated high signal in lesions limited to the bilateral globus pallidus and substantia nigra (Fig. 2a). The patient received supportive and physical therapy. He could walk without support at age 3. Comparison of MR images obtained at 1 year after onset and 5.5 years after onset showed that the basal ganglia and substantia nigra lesions had almost disappeared (Fig. 2b and c). During 8.5 years of follow-up, no recurrent episodes occurred. His performance in school was normal. His growth was in the 90th percentile (height, 134 cm; weight, 26 kg; and head circumference, 54 cm). He had an unsteady gait (Additional file 1: Video S1), mild spasticity of the lower limbs, and brisk symmetrical tendon reflexes. Ophthalmic examination results were normal. Mental development measured using the Wechsler Mental Development Scale-Revised showed a borderline level of mental development, with scores in the 90th percentile.

### Case 2

**Fig. 3:**
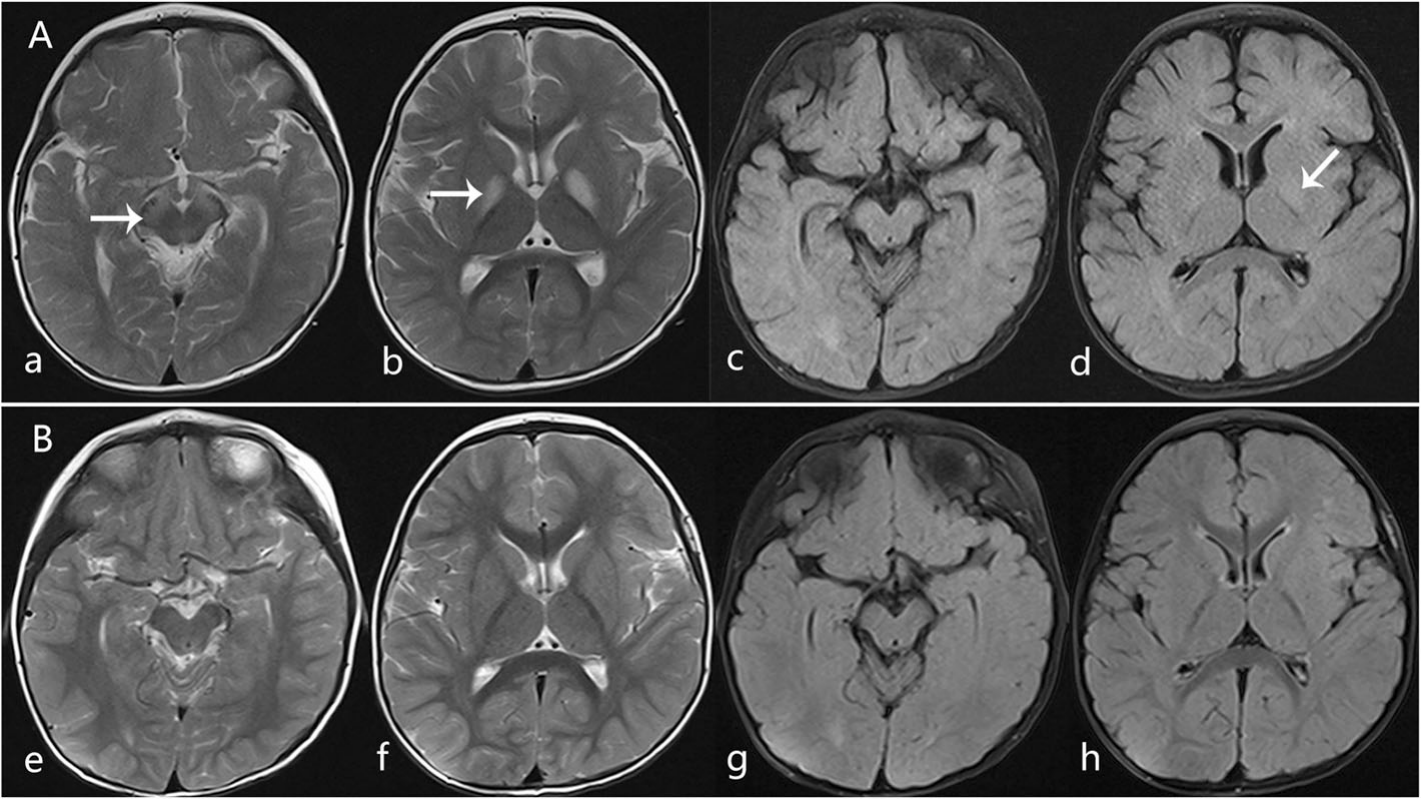
Axial T2WI and FLAIR MRI scans from Case 2. **a-d**, MRI abnormalities observed 2 days after disease onset at 14 months. T2WI (**a**, **b**) and FLAIR (**c**, **d**) showed high signal in the bilateral globus pallidus and substantia nigra (arrows). **e**-**h**, Follow-up images 1.5 years after onset revealed that the original lesions on the basal ganglia and substantia nigra had almost disappeared

This patient was the couple’s second child. She developed lethargy and somnolence and experienced one generalized brief seizure at 14 months after a 4-day episode of mild diarrhea. Her alertness was mildly decreased. Neurologic examination revealed severe generalized hypotonia and an inability to control her head movement. Cranial nerve examination was normal. She had normal deep tendon reflexes. Her blood lactate level was 5 mmol/L, and plasma uric acid was 269 μmol/L. The CSF test results indicated that cell counts, protein levels, and glucose levels were normal. T2-WI and FLAIR brain MRI scans revealed high signal lesions limited to the bilateral globus pallidus and the substantia nigra (Fig. 3a). She reacquired head control at age 18 months. Repeat MRI of the brain was performed at 1.5 years after onset. The results showed that the original lesions had shrunk significantly (Fig. 3b). At 5.5 years of age, she could walk several steps with an unsteady gait. She had some choreiform movements, which became more evident while walking (Additional file 2: Video S2). Her vocabulary was normal (Additional file 3: Video D3). Her growth was in the 90th percentile (height, 110 cm; weight, 18 kg; head circumference, 50 cm). Gesell Developmental Observation-Revised screening was performed to observe her behavior. The results showed a severe delay in gross motor development and a mild delay in language and social-emotional responses. Ophthalmic examination results were normal.

### Case 3

**Fig. 4:**
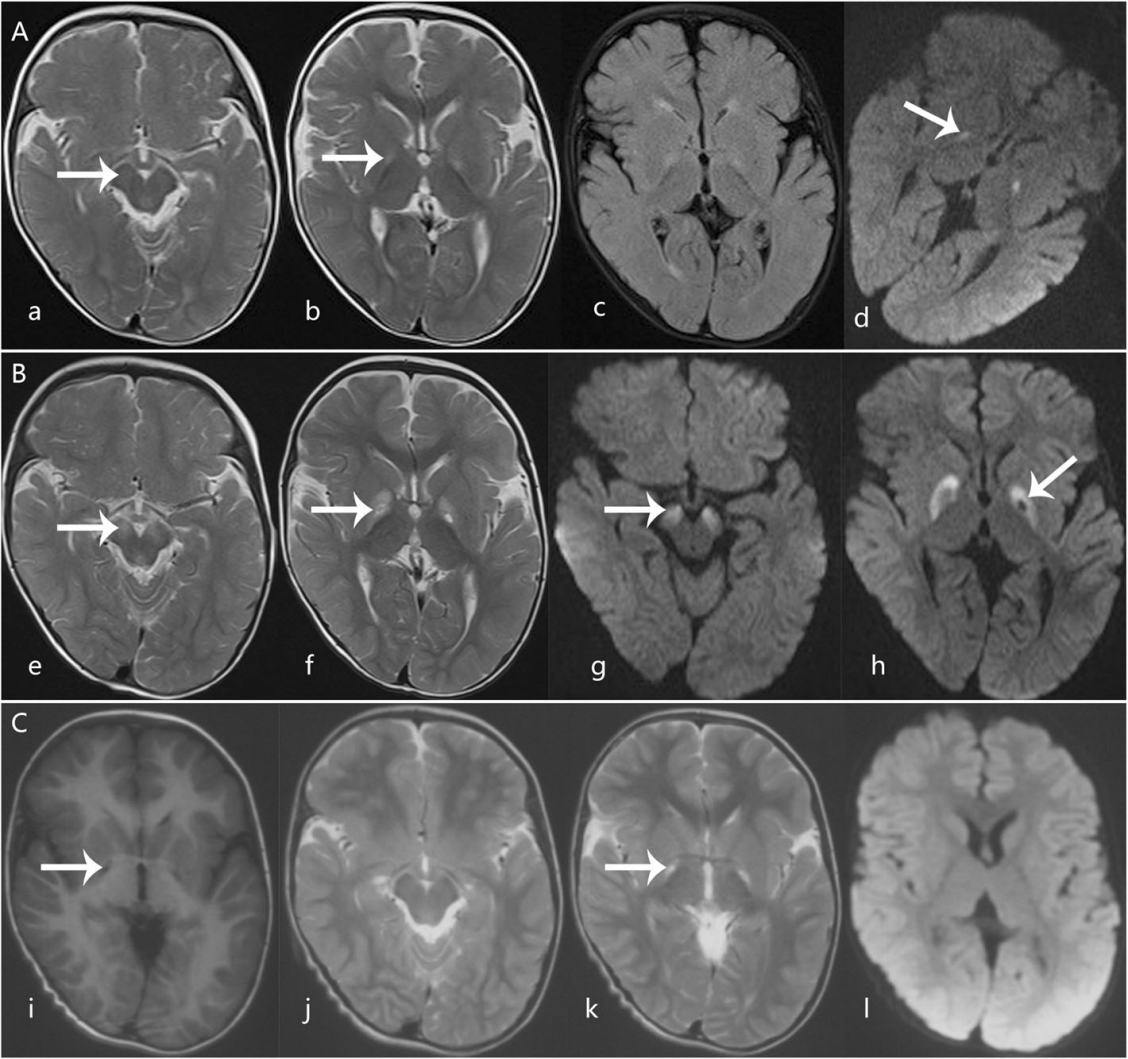
Axial MRI scans from Case 3. T2WI (**a**, **b**), FLAIR (**c**), and diffusion-weighted imaging (DWI) (**d**) performed at 3 days after disease onset at age 16 months showing high signal in the bilateral globus pallidus and substantia nigra (arrows). e-h, Follow-up images 20 days after clinical onset revealed more conspicuous lesions than the original scan, with a necrotic lesion on the left globus pallidus on T2-weighted (**e**, **f**) and diffusion-weighted imaging (**g**, **h**)(arrows). **i**-**l**, Follow-up images at 2.5 years after onset revealed that lesions on the substantia nigra disappeared (**j**) and lesions on the globus pallidus were smaller, with well-delineated cysts (**i**, **k**) (arrows) and without new lesions and brain atrophy

At age 16 months, the second male child was hospitalized due to acute onset of regression of motor and mental skills and choreoathetoid movements. We did not find any prodromal illness prior to the onset of disease. The neurologic examination revealed generalized hypotonia with tendon hyperreflexia and an inability to sit and roll over. Cranial nerve examination findings were normal. T2WI and FLAIR brain MRI scans revealed high signal in the bilateral globus pallidus and substantia nigra. Diffusion-weighted imaging through the basal ganglia revealed hyperintensity of the globus pallidus (Fig. 4a). The patient’s blood lactate level was 2.48 mmol/L, and his homocysteine level was 2.48 μmol/L. Other routine biological parameters, including uric acid levels, were normal. CSF test results were unremarkable (Table1). Symptomatic and supportive therapies improved his symptoms. He was readmitted to the hospital due to a deterioration in movement control following a mild upper respiratory infection 11 days after discharge (20 days after disease onset). Repeat brain MRI revealed additional conspicuous lesions, and T2-weighted and diffusion-weighted imaging uncovered necrotic lesions on the left globus pallidus (Fig. 4b). The patient’s hypotonia progressed to generalized hypertonia over the clinical course. Follow-up MRI scans at 2.5 years after disease onset revealed that the original lesions were smaller (Fig. 4c). Head control was reacquired at age 2. The patient could not sit until 4.5 years of age because of generalized hypertonia. He had some mild choreiform movements (Additional file 4: Video S4). He could only speak a few words but had good language comprehension. His growth was in the 90th percentile (head circumference, 46 cm; height, 100 cm; and weight, 15 kg). Gesell Developmental Observation-Revised screening was performed, and the results showed a severe delay in gross motor development, in fine motor quotient, and a moderate delay in language and social-emotional responses. The patient’s ophthalmic examination results were normal.

### Genetic findings

Physicians suspected Leigh syndrome for Case 1. Genetic sequencing did not reveal the 3243A > G, 8344A > G, 8993A > G, and 8993 T > C mutations. Furthermore, although Case 2 was suspected as succinic semialdehyde dehydrogenase deficiency, genetic sequencing showed no succinic semialdehyde dehydrogenase gene mutation. Therefore, whole-exome sequencing analysis was performed for this family when the eldest child was 9 years of age (the second child was 5 years old, and the third child was 4 years old). Whole-exome sequencing showed that all three siblings harbored two novel compound heterozygous mutations (NM_000456; c.1096C > T [p.R366C] and c.1376G > A [p.R459Q]) in the *SUOX* gene. We confirmed that c.1096C > T came from the maternal line and that c.1376G > A came from the paternal line by Sanger sequencing (Fig. 1b). These mutations are likely pathogenic according to the guidelines for sequence variants provided by the American College of Medical Genetics and Genomics [12]. Residues R366and R459 of *SUOX* are conserved during evolution (Fig. 1c). Three prediction programs (PolyPhen2, SIFT and Mutation Taster) predicted that the mutations could affect the function of SUOX.

*SUOX* is associated with ISOD, and therefore, the levels of sulfite in the urine and plasma homocysteine were measured for each of the three children. This indicated that sulfite concentrations were higher than normal and that homocysteine levels were lower than normal in all three children (Table 1). The three siblings were eventually diagnosed with ISOD. Dietary therapy was not used due to the delayed diagnosis as well as due to economic limitations.

## Discussion and conclusions

Very few patients present with the mild form of ISOD. Previously, only seven patients with mild ISOD have been reported in the literature [7–11, 13]. The clinical features of these patients, including our cases, are listed in Table 2. We have summarized several characteristics of late-onset mild ISOD: older age of onset (mean age, 12 months); seizures (1/10) and ectopia lentis (3/10) are rarely observed; normal development before disease onset (10/10); monophasic clinical course (9/10); and patients are more prone to bilateral globus pallidus and/or substantia nigra involvement (6/10).

**Table 2:** Summary of cases with late-onset mild ISOD

Reference	Case 1	Case 2	Case 3	Shih VE et al., 1977 [7]	Van der Klei-Van et al., 1991 [13]	Barbot et al., 1995 [8]	TouatiG et al., 2000 [9]	TouatiG et al., 2000 [9]	Del RizzoM et al., 2013 [10]	Rocha et al.,2014 [11]
Age of onset/sex	12 months /male	14 months /female	16 months /male	17 months /male	11 months /male	3 months /female	15 months /male	8 months /female	12 months /female	12 months /female
Perinatal period/ family history	Normal/ unremarkable	Normal/ unremarkable	Normal/ unremarkable	Normal/ unremarkable	Normal/ unremarkable	Normal/ unremarkable	Normal/ unremarkable	Normal/ a elder sister with ISOD	Normal/ unremarkable	Normal/ unremarkable
Age in report (year)	9.5	5.5	4.5	6.5	1	7	4 years and 5mon	3 years and 2mon	2 years and 6 mon	4
Clinical features	Rapid regression of acquired motor skills and cognition, dystonia	Poor response and experienced one generalized brief seizure, dystonia	Regression of motor and mental skills and choreoathetoid movements, dystonia	Psychomotor retardation, choreiform movement of right side of body	Psychomotor retardation,choreiform movement	Choreoathetoid movements, lost transiently headcontrol	Choreoathetoid movements, inability to walk, hyperkinesia	Slight motor delay, moderate axial hypotonia	Psychomotor retardation,acute left hemiparesis, generalized mild hypotonia	Psychomotor retardation,trunk and gait ataxia, generalized hypotonia
Ectopialentis (year)	No ectopialentis (9.5)	No ectopialentis (5.5)	No ectopialentis (4.5)	Yes (4)	NA	No ectopialentis (7)	No ectopialentis (4 years and 5 months old)	Yes (8 months)	NA	Yes (3 years and 10 months)
Laboratory finding										
Urine sulfite test (normal values)	45, 50 (< 15)	30, 40 (< 15)	25, 100 (< 15)	13.8(ND)	12(ND)	P (negative)	P (negative)	P (negative)	30(ND)	P (negative)
S-sulfocysteine plasma/urine (normal values)	No	No	No	26 (0)/NA	24(ND)/1.7 (0–0.2)	20 (0)/220 (0)	stronglyelevated	20 (0)/NA	28 (0)/313 (0)	141(0–0.1)/NA
Plasma total homocysteine	3.74 (normal 5–15)	3.17 (normal 5–15)	2.48, 2.66 (normal 5–15)	NA	NA	NA	NA	NA	< 1(normal> 4)	0.6 (normal> 4)
Uric acid (μmol/L)	Normal	Normal	Normal	NA	175(normal)	Normal	148 (normal)	220 (normal)	NA	NA
Sulfite oxidase activity	No	No	No	No activity was detected	Totally absent	Completely absent	ND	ND	ND	ND
Neuroimaging findings	MRI, hyperintense signal of bilateral globus pallidi, and substantia nigra	MRI, hyperintense signal of bilateral globus pallidi, and substantia nigra	MRI, hyperintense signal of bilateral globus pallidi, and substantia nigra	NA	Head computed tomography, no abnormalities	MRI, symmetrical involvement of the globus pallidus, cerebello medullary enlarged	MRI, hypodensity of the white matter and frontal lobes	NA	MRI, mild cerebral atrophy and asymmetric stroke-like lesions of the globus pallidus	MRI, hyperintense signal of bilateral globus pallidi, together with cerebral peduncle involvement
Gene test results										
Nucleotide, protein	c.1096C > T, p.R366C; c.1376G > A, p.R459Q	c.1096C > T, p.R366C; c.1376G > A, p.R459Q	c.1096C > T, p.R366C; c.1376G > A, p.R459Q	No	No	No	No	No	c.427C > A, p.H143N	c.182 T > C; p.L61P
Domain	Homodimerization and Moco domain	Homodimerization and Moco domain	Homodimerization and Moco domain	No	No	No	No	No	Homodimerization	Transit peptide
Dietary treatment (duration)	No	No	No	Yes (5 years)	NA	Yes (2 weeks)	Yes (2 years)	Yes (2 years)	Yes(18 months)	Low protein diet (NA)
Outcomes	His performance in school was normal. He had unsteady gait follow up till age of 9.5	She could walk several steps without support with an unsteady gait. Her vocabulary was normal follow up till age of 5.5	He could only speak a few words but had good language comprehension follow up till age of 4.5	Improvement in biochemical and clinical results	NA	Slowly progressive neurology disorder with ataxic gait, dystonia, and choreoathetoid movements	Became much more calm and less aggressive, and started to talk	Progressing well. She walked alone at 21 months, and started to speakat 2 years	Biochemicalimprovement was observed with progressive clinical amelioration	Slight truncal ataxia. No further episodes were observed over the next thirteen months

Abbreviations are as follows: *ISOD* isolated sulfite oxidase deficiency, *MRI* magnetic resonance imaging, *NA* not available, *UD* undetectable, *No* not performed, *P* positive

Mechanisms for milder presentations of ISOD have not been fully elucidated. It has been reported that patients with missense mutations in the *SUOX* gene have a milder clinical presentation than those with null mutations [2], since missense mutations of the *SUOX* gene only lead to decreased biosynthesis of SUOX, whereas null mutations of the *SUOX* gene lead to complete abolishment of SUOX biosynthesis [11]. The genetic data from the case series presented here and from 2 cases of mild ISOD reported by others indicated the presence of missense mutations in the *SUOX* gene, further supporting the hypothesis that missense mutations of the *SUOX* gene contribute to a milder presentation.

Our case series and 2 cases of late-onset mild ISOD reported by others show decreased homocysteine levels. Sulfite reacts readily with free thiol groups to form sulfocysteine. It can also conjugate with homocysteine to form sulfocysteine, thus depleting plasma homocysteine [2]. Therefore, decreased homocysteine levels may facilitate a diagnosis of ISOD [11]. The slightly decreased levels of homocysteine were ignored by our clinicians before the results from exome sequencing for this family were made available. Therefore, we suggest that physicians need to be aware that decreased levels of homocysteine are observed in late-onset mild ISOD.

More than 90% of patients with classic early-onset ISOD show severe cerebral and cerebellar atrophy and/or cystic white matter changes [2, 14, 15]. However, brain MRI scans of mild cases, including the three cases reported here, showed lesions mainly in the bilateral globus pallidus and substantia nigra at the acute stage, and later, the lesions improved and stabilized, suggesting that this pattern of bilateral globus pallidus and/or substantia nigra damage might be specific to mild ISOD. This imaging pattern may explain why patients in this cohort mainly present with motor regression and movement disorders rather than seizures and developmental delays. Neuro-imaging patterns of the symmetric basal ganglia and the brain stem have etiological diversity in children. Similar neuro-imaging findings are observed both in inherited metabolic diseases and acquired disorders, including Leigh syndrome, maple syrup urine disease, Wilson disease, organic acidurias, succinic semialdehyde dehydrogenase deficiency, kernicterus, and carbon monoxide poisoning. Most of these inherited metabolic diseases are progressively aggravated over the clinic course, and regular metabolic screening may provide diagnostic indicators. Furthermore, identification of acquired etiologies would be realized in cases of kernicterus and carbon monoxide poisoning. Symmetric basal ganglia involvement with low plasma homocysteine with or without dislocated lens would be important clues for the diagnosis of ISOD, especially the late-onset mild form.

Restricting dietary intake of methionine, cysteine, and taurine has been reported to be effective in patients with mild ISOD [7–11]. Touati et al. reported that when two children in a family were affected, the prognosis of the child who used dietary therapy was better than that of the child who did not [9]. However, clinical and neuroimaging improvements were observed in our patients even though dietary therapy was not used, suggesting that late-onset mild ISOD might spontaneously recover in some circumstances. Future studies to elucidate the mechanistic link between the genotype and phenotype of the disease and outcomes of patients with ISOD are required to gain better insight.

In conclusion, to our knowledge, this is the first literature review to summarize the characteristics of late-onset mild ISOD and the first report of late-onset mild ISOD cases with a stable clinical course and spontaneous recovery without dietary therapy. We propose that when patients present with late-onset symptoms, a monophasic clinical course, neuroimaging indicating a bilateral globus pallidus lesion, and decreased homocysteine levels, ISOD should be considered. Furthermore, we can reasonably predict a good prognosis for children with late-onset mild ISOD based on their monophasic clinical course and reversible neuroimaging features.

## Supplementary information

**Additional file 1: Video S1.** Video for case 1 at the age of 9.5 years showing the movement ability. The boy had unsteady gait, no choreiform movements were observed.

**Additional file 2: Video S2.** Video for case 2 at the age of 5.5 years showing the movement ability The girl could walk several steps without support, with an unsteady gait and choreiform movements.

**Additional file 3: Video S3.** Video for case 2 at the age of 5.5 years showing the language ability. Her vocabulary was normal, she could read smoothly.

**Additional file 4: Video S4.** Video for case 3 at the age of 4.5 years showing the movement ability. He could not sit until age 4.5 years because of generalized hypertonia. He had some mild choreiform movements.

## Data Availability

All data generated or analyzed during this study are included in this article.

## Abbreviations

CSF: Cerebrospinal fluid
FLAIR: Fluid-attenuated inversion recovery
ISOD: Isolated sulfite oxidase deficiency
MRI: Magnetic resonance imaging
*SUOX*: Sulfite oxidase gene
SUOX: Sulfite oxidase protein
T2WI: T2-weighted
